# The Identification of Childhood Asthma Progression-Related lncRNAs and mRNAs Suitable as Biomarkers Using Weighted Gene Coexpression Network Analysis

**DOI:** 10.1155/2021/5511507

**Published:** 2021-07-27

**Authors:** Min Hao, Jinling Zan

**Affiliations:** ^1^Department of Pediatrics, Zaozhuang Municipal Hospital, Zaozhuang, Shandong 277100, China; ^2^Department of Intensive Care Unit, Zaozhuang Municipal Hospital, Zaozhuang, Shandong 277100, China

## Abstract

**Background:**

Asthma is a common chronic respiratory disease in children, seriously affecting children's health and growth. This bioinformatics study aimed to identify potential RNA candidates closely associated with childhood asthma development within current gene databases.

**Methods:**

GSE65204 and GSE19187 datasets were screened and downloaded from the NCBI GEO database. Differentially expressed long noncoding RNAs (DE-lncRNAs) and mRNAs (DE-mRNAs) were identified using the Bioconductor limma package in R, and these DE-mRNAs were used to perform biological process (BP) and Kyoto Encyclopedia of Genes and Genomes (KEGG) analyses. Thereafter, weighted gene coexpression network analysis (WGCNA) was utilized to screen the modules directly related to childhood asthma, and a coexpression network of DE-lncRNAs and DE-mRNAs was built. Finally, principal component analysis (PCA) was performed.

**Results:**

In total, 7 DE-lncRNAs and 1060 DE-mRNAs, as well as 7 DE-lncRNAs and 1027 DE-mRNAs, were identified in GSE65204 and GSE19187, respectively. After comparison, 336 overlapping genes had the same trend of expression, including 2 overlapped DE-lncRNAs and 334 overlapped DE-mRNAs. These overlapped DE-mRNAs were enriched in 28 BP and 12 KEGG pathways. Eleven modules were obtained in GSE65204, and it was found that the purple, black, and yellow modules were significantly positively correlated with asthma development. Subsequently, a coexpression network including 63 DE-mRNAs and 2 DE-lncRNAs was built, and five KEGG pathways, containing 8 genes, were found to be directly associated with childhood asthma. The PCA further verified these results.

**Conclusion:**

LncRNAs *LINC01559* and *SNHG8* and mRNAs *VWF*, *LAMB3*, *LAMA4*, *CAV1*, *ALDH1A3*, *SMOX*, *GNG4*, and *PPARG* were identified as biomarkers associated with the progression of childhood asthma.

## 1. Introduction

Asthma is one of the most common chronic inflammatory respiratory diseases worldwide. It is caused by a combination of genetic, environmental, and lifestyle factors and is characterized by inflammation, airway hyperresponsiveness, and variable airflow obstruction [1]. Asthma not only affects the health and life of adults but also seriously influences the physical and mental health of children at the growth stage. Approximately one-third to one-half of children with moderate to severe asthma may have it persist into adulthood, which has exerted a heavy burden on the healthcare system [2]. It has been reported that the prevalence, incidence, and mortality of childhood asthma worldwide are increasing year by year [3]. With the current rising trend, childhood asthma is likely to become an increasingly severe public health problem. Currently, corticosteroids, *ß*-agonists, magnesium sulfate, and anticholinergic drugs are the primary medications used for treating childhood asthma [4]. However, these drugs require long-term use, which can contribute to side effects and drug resistance in children. Additionally, herbs, including *Glycyrrhiza glabra* (licorice), have also been used as medicines to treat respiratory diseases such as asthma [5]. However, the specific treatment mechanism remains unclear. Despite its prevalence and global impact, the physiological and pathological mechanisms underlying the occurrence and progression of childhood asthma remain elusive. This has motivated this study and its search for novel biomarkers for the diagnosis of and therapeutic strategies for asthma.

Long noncoding RNAs (lncRNAs) are a class of noncoding RNAs with a length greater than 200 nt and are regulators of various biological processes, including chromatin structure changes, transcriptional activation/inhibition, intracellular trafficking, and microRNA (miRNA) chelation and translation efficiency regulation [6, 7]. Although the roles lncRNAs may play in disease development have not been fully elucidated, lncRNAs have been reported to participate in the development of cancer due to their function as a gene regulator. LncRNAs have been suggested as potential biomarkers for many diseases, such as hepatocellular carcinoma [8], melanoma [9], and cardiovascular diseases [10]. A previous study by Wei et al. [11] showed that lncRNA *MEG3* was highly expressed in healthy tissues adjacent to gastric cancer tissues and that its overexpression could inhibit the proliferation and metastasis of gastric cancer cells through the p53 signaling pathway. Cao et al. [12] reported that lncRNA *RMRP* was significantly upregulated in bladder cancer tissues and was closely related to tumor size, lymph node metastasis, and survival time of patients. LncRNA *RMRP* can promote the proliferation, migration, and invasion of bladder cancer cell lines [12]. All these research studies indicated that lncRNAs are involved in the occurrence and development of various cancers. Furthermore, Dolcino et al. [13] analyzed the transcript profiles of rheumatoid arthritis (RA) patients and healthy donors and found that lncRNA RP11-4989.15 plays an important role in RA pathogenesis. Another study screened eight important lncRNAs interacting with 52 differentially expressed genes enriched in asthma-associated pathways, and these lncRNAs have the potential to serve as underlying biomarkers useful for the future development of therapeutic strategies [14]. Nevertheless, specific biomarkers for childhood asthma have not been identified, and the roles of lncRNAs in childhood asthma remain unclear.

Weighted gene coexpression network analysis (WGCNA) has been successfully used to identify highly correlated gene clusters (coexpression modules) and to identify potential biomarkers or therapeutic targets significantly associated with clinical phenotypes [15, 16]. Zhao and Fan [17] successfully used WGCNA and univariate Cox regression analysis methods to identify five lncRNAs (*GAS5*, *HCP5*, *PART1*, *SNHG11*, and *SNHG5*) to predict the prognosis of ovarian cancer. Therefore, this study aims to identify differentially expressed RNAs (DERs) closely related to asthma progression in children using WGCNA. These findings may provide novel diagnostic biomarkers and therapeutic targets for the development of childhood asthma.

## 2. Materials and Methods

### 2.1. Data Source

On November 5, 2020, datasets that met the following criteria were screened in the NCBI GEO database (https://www.ncbi.nlm.nih.gov/) [18] with the search keywords “asthma, child, childhood” and the following search restrictions: (1) the samples are from the same type (both solid or blood or tissue samples); (2) the samples are classified as disease and control groups; (3) the total number of samples included in the analysis is more than 20, and each type has at least two duplicate samples. Two sets of available data, GSE65204 [19] and GSE19187 [20], were screened and downloaded. GSE65204 contained 69 samples of nasal epithelial tissue from inner-city children aged 10–12 y, including 33 healthy samples and 36 asthma samples. GSE19187 consisted of 11 healthy samples and 13 asthma samples from children aged 6–17 y. The detection platforms used for the samples in GSE65204 and GSE19187 were Agilent-028004 SurePrint G3 Human GE 8x60K Microarray and Affymetrix Human Gene 1.0 ST Array, respectively.

### 2.2. Differentially Expressed RNA (DER) Screen

The detailed annotation information of the platforms was downloaded from Ensembl genome browser, release 96 (https://asia.ensembl.org/index.html), including annotation files, transcript IDs, and RefSeq IDs. The expression profiles in GSE65204 and GSE19187 were reannotated into mRNAs and lncRNAs.

The Bioconductor limma package (version 3.34.0, https://bioconductor.org/packages/release/bioc/html/limma.html) compiled in R 3.6.1 [21] was used to identify differential gene expression profiles of DERs in both GSE65204 and GSE19187 datasets, including differentially expressed mRNAs (DE-mRNAs) and differentially expressed lncRNAs (DE-lncRNAs). The threshold values for selecting DERs were a false discovery rate (FDR) of <0.05 and |log_2_ fold change (FC) |>0.5. By comparing DERs between GSE65204 and GSE19187, the DERs with the same differential expression were chosen as the overlapped DERs (overlapped DE-lncRNAs and DE-mRNAs) related to asthma. These screened overlapped DE-mRNAs were utilized to perform biological process of Gene Ontology (GO BP) and Kyoto Encyclopedia of Genes and Genomes (KEGG) pathway enrichment analyses using DAVID version 6.8 (https://david.ncifcrf.gov/) [22]. A *P* value of <0.05 was used as the cutoff criterion for enrichment significance.

### 2.3. Screen of the Significantly Stable Modules Related to Asthma by WGCNA

The WGCNA package (version 1.61, https://cran.r-project.org/web/packages/WGCNA/index.html) compiled in R [23] was used to screen the significantly stable modules related to asthma. GSE65204 contained a relatively large number of samples and served as the training set, and GSE19187 was instead used as the validation set. The parameters were set as follows: (1) ≥100 RNAs were included in one module; (2) cutHeight = 0.995. Subsequently, the overlapped DE-mRNAs were mapped to each module from WGCNA, and then the fold enrichment parameter and *Pp* value of the DE-mRNAs were calculated using a hypergeometric algorithm [24]. *P* < 0.05 and fold enrichment >1 served as the thresholds for module filtering. The formula of the hypergeometric algorithm was (*kNMn*)=*C* (*kM*) × *C*(*n* − *k*, *N* − *M*) ÷ *C*(*nN*), where *N* denotes all genes involved in WGCNA analysis, *M* denotes the number of genes in each module, *n* denotes the number of overlapping DE-mRNAs, and *k* represents the number of DE-mRNAs mapped to the corresponding module.

### 2.4. Coexpression Network Construction

The Pearson correlation coefficient (PCC) between DE-mRNAs enriched in the modules and overlapped DE-lncRNAs from the earlier differential expression screen was calculated using the cor function in R (https://77.66.12.57/R-help/cor.test.html). Subsequently, a coexpression network was constructed, and Cytoscape 3.6.1 (https://www.cytoscape.org/) was used for visualization [25]. Afterwards, the DAVID tool was utilized to analyze the KEGG pathway enrichment of the genes in the coexpressed network (*P* < 0.05).

### 2.5. Identification of RNAs Directly Related to Asthma and Principal Component Analysis (PCA)

With “asthma” as the keyword, Comparative Toxicogenomics Database (2019 update) (https://ctd.mdibl.org/) [26] was utilized to search the KEGG pathways directly associated with asthma. By comparing pathways after searching to those in the coexpressed network, we used the overlapping pathways to identify the important genes directly related to asthma.

PCA is a dimension reduction technique that transforms multiple variables into a few principal components that can reflect most of the information of the original variables [27]. PCA was carried out on these identified asthma-related genes using the Personality Project Psych package version 2.1.6 (https://CRAN.R-project.org/package=psych) compiled in R.

## 3. Results

### 3.1. Identification of DE-lncRNAs and DE-mRNAs

**Figure 1:**
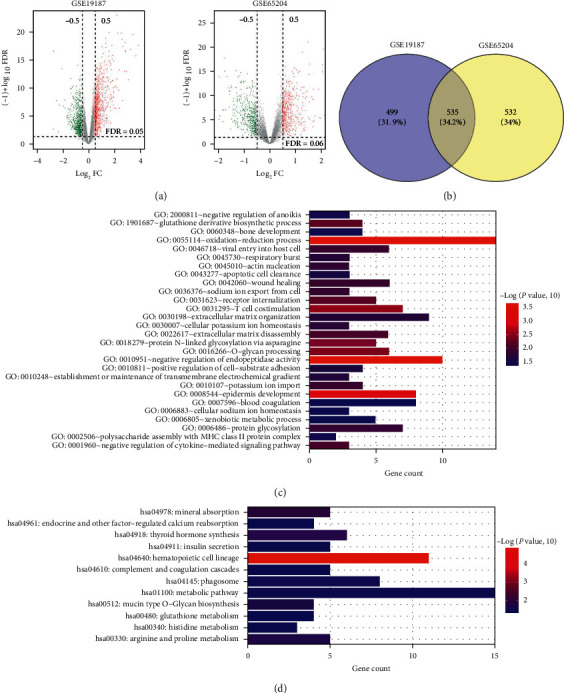
Identification of differentially expressed RNAs (DERs) and functional analyses of overlapped differentially expressed mRNAs (DE-mRNAs). (a) The volcano figures of DERs. Left: the volcano figure of DERs in the GSE19187 dataset. Right: the volcano figure of DERs in the GSE65204 dataset. Green and red dots represent downregulated and upregulated DERs, respectively. The horizontal dotted line represents the value of the false discovery rate <0.05; the vertical dotted line represents the value of the |log_2_ fold change (FC)| > 0.5. (b) Venn diagram of DERs found in the GSE19187 and GSE65204 datasets and DERs common between them. (c) Biological processes (BPs) significantly associated with overlapped DE-mRNAs. (d) The overlapped DE-mRNAs significantly enriched in Kyoto Encyclopedia of Genes and Genomes (KEGG) pathways.

After reannotation and processing, 135 lncRNAs and 11,808 mRNAs were obtained. Afterwards, DEGs between normal samples and asthma samples were identified. In total, there were 1067 DERs (7 DE-lncRNAs and 1060 DE-mRNAs) found in GSE65204 and 1034 DERs (7 DE-lncRNAs and 1027 DE-mRNAs) found in GSE19187 (Figure 1(a)). By comparing the DERs between the two datasets, 535 overlapping genes were found (Figure 1(b)), but only 336 overlapping genes had the same differential expression, including 2 overlapped DE-lncRNAs (*SNHG8* and *LINC01559*) and 334 overlapped DE-mRNAs.

### 3.2. Functional Enrichment Analyses of Overlapped DE-mRNAs

These 334 overlapped DE-mRNAs were used for GO BP and KEGG analyses. It was found that these DE-mRNAs were enriched in 28 biological processes, such as “negative regulation of endopeptidase activity,” “oxidation-reduction process,” “epidermis development,” “T-cell costimulation,” “O-glycan processing,” “protein N-linked glycosylation via asparagine,” “glutathione derivative biosynthetic process,” “extracellular matrix organization,” and “bone coagulation” (Figure 1(c)). Additionally, these overlapped DE-mRNAs were enriched into 12 KEGG pathways: “hematopoietic cell lineage,” “mineral absorption,” “thyroid hormone synthesis,” “arginine and proline metabolism,” “metabolic pathways,” “mucin-type O-glycan biosynthesis,” “phagosome,” “complement and coagulation cascades,” “endocrine and other factor-regulated calcium reabsorption,” “histidine metabolism,” “glutathione metabolism,” and “insulin secretion” (Figure 1(d)).

### 3.3. Significantly Stable Asthma-Related Modules Identified by WGCNA

**Figure 2:**
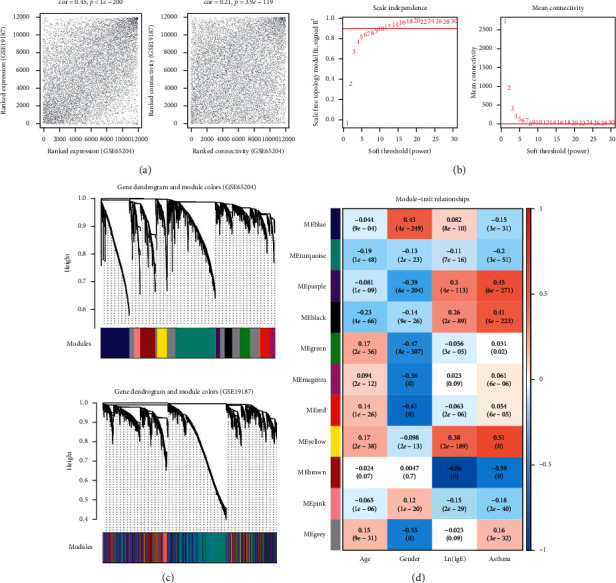
Screen of significantly stable modules related to asthma in children by weighted gene coexpression network analysis (WGCNA). (a) The correlation of gene expression levels (left) and network connections (right) between the training set (GSE65204) and the validation set (GSE19187). (b) Left: adjacency matrix weight parameter power selection graph. The red line represents the standard line where the square value of the correlation coefficient reaches 0.9. Right: schematic diagram of the average connectivity of DE-mRNAs under different power parameters. The red line represents the value of the average degree of the coexpression network (=1) under the value of the weight parameter power of the adjacency matrix in the left. (c) The module classification trees of genes in GSE65204 (above) and GSE19187 (below). Each color represents a different module. (d) The heat map of correlations between clinical information of samples and each module obtained from the training set.

WGCNA was performed on all DERs in GSE65204 and GSE19187, using GSE65204 as the training set and GSE19187 as the validation set. To ensure that the gene expression levels in each dataset were comparable, we first analyzed the expression level and connectivity consistency of all gene expression values detected in these two datasets. It was found that the gene expression levels and network connections were significantly positively correlated between the training and validation sets, which indicated that the two datasets were indeed comparable (Figure 2(a)). We subsequently identified significantly stable asthma-related modules in the two datasets. Based on GSE65204, the power value was 12, and the average degree of the coexpression network was 1 (Figure 2(b)). Hierarchical cluster trees were then generated from both datasets (including 11 modules from GSE65204) (Figure 2(c)). The heat map of correlations between the clinical information of samples and each module obtained from the training set is displayed in Figure 2(d). The results showed that the purple, black, and yellow modules were significantly positively correlated with asthma development (Figure 2(d)).

Then, the stability of the modules was assessed. By analyzing, it was found that there were 6 significantly stable modules with a preservation *Z*-score higher than 5 (Table 1). According to the hypergeometric algorithm, the above 334 overlapped DE-mRNAs were mapped to each module identified by WGCNA, and a total of 242 overlapping genes were found. Two of the modules (black and yellow) were significantly enriched, with 14 black and 58 yellow consistent DE-mRNAs found. The 72 consistent DE-mRNAs were utilized for further study.

### 3.4. Establishment of the Coexpression Network

**Figure 3:**
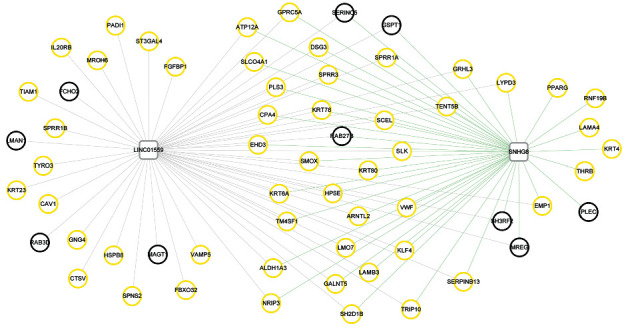
LncRNA-mRNA coexpression networks. The squares and circles represent lncRNAs and mRNAs, respectively. The color of the border of the circles represents the color of the corresponding WGCNA modules.

PPCs between the 72 consistent DE-mRNAs found in the previous analysis and the aforementioned 2 overlapped DE-lncRNAs were calculated. The connection pairs between mRNAs and lncRNAs were retained with PCC >0.4. Subsequently, a coexpression network, including 2 DE-lncRNAs and 63 DE-mRNAs, was generated using these connection pairs (Figure 3). KEGG pathway analysis found that these DE-mRNAs were significantly enriched in five KEGG pathways: “ECM-receptor interaction,” “focal adhesion,” “beta-alanine metabolism,” “PI3K-Akt signaling pathway,” and “pathways in cancer” (Table 2).

A total of 195 KEGG pathways directly related to asthma were obtained from the CTD database. After comparison with the DE-mRNA-enriched pathways in the coexpression network, the five enriched KEGG pathways (containing eight genes) were shown to be directly associated with asthma (Table 2). It is clear that the von Willebrand factor (*VWF*), laminin subunit beta 3 (*LAMB3*), and laminin subunit alpha 4 (*LAMA4*) genes were involved in “ECM-receptor interaction,” “focal adhesion,” and “PI3K-Akt signaling pathway,” while *LAMB3* and *LAMA4* were also involved in “pathways in cancer.” In addition, caveolin-1 (*CAV1*) was involved in “focal adhesion,” and aldehyde dehydrogenase 1 family member A3 (*ALDH1A3*) and spermine oxidase (*SMOX*) were associated with “beta-alanine metabolism.” G protein subunit gamma 4 (*GNG4*) was related to the “PI3K-Akt signaling pathway” and “pathways in cancer.” Additionally, peroxisome proliferator-activated receptor gamma (*PPARG*) was involved in “pathways in cancer.” The coexpression network found that lncRNA *LINC01559* may be coexpressed with *VWF*, *LAMB3*, *CAV1*, *ALDH1A3*, *SMOX*, and *GNG4*. lncRNA *SNHG8* might coexpress with *VWF*, *LAMB3*, *LAMA4*, *ALDH1A3*, *SMOX*, and *PPARG*. This analysis suggests that these lncRNAs, mRNAs, and pathways may be closely associated with childhood asthma development.

### 3.5. PCA

**Figure 4:**
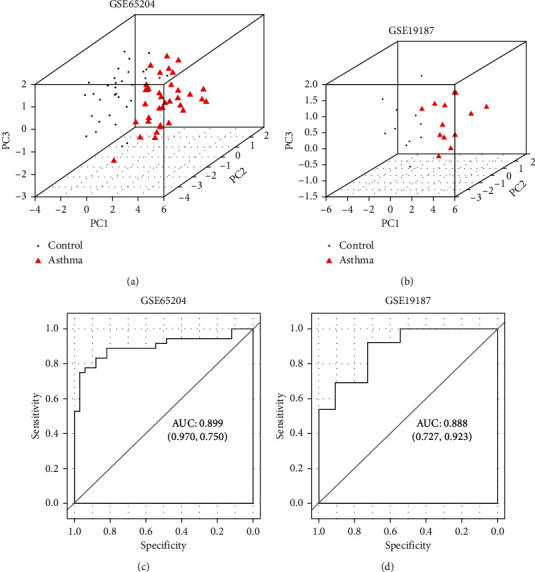
Principal component analysis (PCA) of the identified genes in the coexpression network. Three-dimensional diagrams of the sample distribution based on PC1, PC2, and PC3 in GSE65204 (a) and GSE19187 (b). The black dots represent the healthy samples, and the red triangles represent the asthma samples. The *X*-, *Y*-, and *Z*-axes represent PC1, PC2, and PC3, respectively. Receiver operating characteristic (ROC) curves in GSE65204 (c) and GSE19187 (d). The figures in brackets represent specificity and sensitivity, respectively.

PCA was performed on the eight genes involved in the five asthma-related pathways. Through the calculation and analysis of gene expression values in GSE65204 and GSE19187, a total of eight principal components (PCs) were obtained by fitting. Among them, the cumulative contribution rate of PC1, PC2, and PC3 was over 80%, which implied that these three PC factors contained significant information about the original variables (gene expression values). Thereafter, PC1, PC2, and PC3 were used to construct three-dimensional diagrams of the sample distribution and to build receiver operating characteristic (ROC) curves. As shown in Figures 4(a) and 4(b), PC1, PC2, and PC3 could significantly distinguish between the control and asthma samples. The area under the curve (AUC) of the ROC curves in GSE65204 and GSE19187 was 0.899 and 0.888, respectively (Figures 4(c) and 4(d)). These results further demonstrate that *VWF*, *LAMB3*, *LAMA4*, *CAV1*, *ALDH1A3*, *SMOX*, *GNG4*, and *PPARG* are closely associated with the occurrence and development of childhood asthma.

## 4. Discussion

Asthma is a common chronic respiratory disease in children, seriously affecting children's health and growth [28]. Since the etiology of childhood asthma is complex and its pathogenesis is still unclear, future research must focus on exploring potential diagnostic and therapeutic biomarkers for disease progression. This study identified a total of 1067 DERs (7 DE-lncRNAs and 1060 DE-mRNAs) and 1034 DERs (7 DE-lncRNAs and 1027 DE-mRNAs) in the GSE65204 and GSE19187 datasets, respectively. After comparison between GSE65204 and GSE19187, two overlapped DE-lncRNAs (*SNHG8* and *LINC01559*) and 334 overlapped DE-mRNAs had the same differential expression. After that, significantly stable modules related to asthma were identified by WGCNA, and a coexpression network was built with 2 DE-lncRNAs and 63 DE-mRNAs. After functional analysis of genes in the coexpression network, five KEGG pathways (“ECM-receptor interaction,” “focal adhesion,” “beta-alanine metabolism,” “PI3K-Akt signaling pathway,” and “pathways in cancer”) contained 8 genes (*VWF*, *LAMB3*, *LAMA4*, *CAV1*, *ALDH1A3*, *SMOX*, *GNG4*, and *PPARG*) which were found to be directly related to childhood asthma development and have potential for use as disease progression biomarkers.

Previous studies have shown that lncRNAs play important roles in the development of pediatric airway diseases, including asthma [29, 30]. Our study found that lncRNAs *SNHG8* and *LINC01559* were found to be associated with the progression of childhood asthma. *SNHG8*, located on 4q26, regulates tumorigenesis and metastasis and controls the progression of multifarious diseases, such as hepatocellular carcinoma [31], pancreatic adenocarcinoma [32], endometrial carcinoma [33], and gastric carcinoma [34]. Wang et al. indicated that *LINC01559* was upregulated in gastric cancer tissues and could stimulate the PI3K-Akt signaling pathway to accelerate the progression of gastric cancer [35]. Therefore, we speculate that lncRNAs *SNHG8* and *LINC01559* may participate in the occurrence and development of childhood asthma by regulating cell proliferation, migration, and PI3K-Akt signaling pathway.

In addition, from the lncRNA-mRNA coexpression network, *LINC01559* might coexpress with *VWF*, *LAMB3*, *CAV1*, *ALDH1A3*, *SMOX*, and *GNG4*, and *SNHG8* might coexpress with *VWF*, *LAMB3*, *LAMA4*, *ALDH1A3*, *SMOX*, and *PPARG*, which were enriched in five KEGG pathways, including “ECM-receptor interaction,” “focal adhesion,” “beta-alanine metabolism,” “PI3K-Akt signaling pathway,” and “pathways in cancer.” PCA further verified that these eight genes are important in childhood asthma. *VWF*, a large plasma glycoprotein, plays a vital role in mediating the balance between blood clotting and bleeding [36]. A study by Yao et al. [37] showed that *VWF* was highly expressed in an IL-25-induced murine asthma model and was associated with bronchial mucosal vascular remodeling in asthma. *LAMB3* and *LAMA4* belong to the laminin subunit family and have been reported to be involved in the metastasis and invasion of some types of cancer [38, 39]. A study by Zhang et al. [40] demonstrated that *LAMB3* could mediate apoptosis, proliferation, migration, and invasion of pancreatic ductal adenocarcinoma cells through the PI3K/Akt signaling pathway. Another study found that high expression of *LAMA4* may be a new marker of tumor invasion and metastasis in human hepatocellular carcinoma [41]. *CAV1* is a carcinogenic membrane protein associated with extracellular matrix tissue, cell migration, cholesterol distribution, endocytosis, and signal transduction [42]. In our study, *CAV1* participated in focal adhesion, thus contributing to the pathogenesis of childhood asthma. *GNG4* is one of the fourteen *γ*-subunit proteins of the G protein trimer complex [43], and Liu et al. [44] found that *GNG4* associated with the PI3K-Akt signaling pathway to participate in rectal cancer through using a bioinformatics approach. PPARG is one of the three subtypes of peroxisome proliferator-activated receptors, and its activation has been reported to regulate the synthesis and release of immunomodulatory cytokines from various cell types [45]. A previous study has shown that PPARG modulates inflammation and may have an effect on regulating the long-term control of asthma in children and young adults [46].

Additionally, *ALDH1A3* and *SMOX* were also essential for childhood asthma and were enriched in the “beta-alanine metabolism” pathway. *ALDH1A3* is widely distributed in normal tissues and abnormally expressed in a variety of cancers [47]. Li et al. [48] indicated that *ALDH1A3* can serve as an activator of mesenchymal differentiation and may be a marker for predicting the survival rate of glioblastoma patients. *SMOX*, a member of the polyamine oxidases, catalyzes the oxidative degradation of polyamine spermidine to produce spermidine and is caused by a variety of stimuli, including bacterial infection and oxidative stress [49]. Jain et al. [50] found low expression of *SMOX* in bronchial epithelial cells (BECs) of asthmatic lung samples, and *SMOX* knockdown resulted in asthma in naive mice, such as airway hyperresponsiveness, remodeling, and BEC apoptosis. Combined with our results, we speculate that lncRNAs *LINC01559* and *SNHG8* may be coexpressed with *VWF*, *LAMB3*, *LAMA4*, *CAV1*, *ALDH1A3*, *SMOX*, GNG4, and *PPARG*, and these lncRNAs and mRNAs may be direct biomarkers related to the occurrence and progression of childhood asthma. However, the relationships between lncRNAs and their target genes need to be further characterized, and use of these identified lncRNAs and mRNAs as biomarkers will require further validation in a clinical setting.

## 5. Conclusion

In conclusion, *LINC01559* and *SNHG8* may be coexpressed with *VWF*, *LAMB3*, *LAMA4*, *CAV1*, *ALDH1A3*, *SMOX*, *GNG4*, and *PPARG*. Additionally, these lncRNAs and mRNAs may be directly involved in the pathogenesis of childhood asthma through ECM-receptor interaction, focal adhesion, beta-alanine metabolism, PI3K-Akt signaling pathway, and pathways in cancer. The two lncRNAs and eight genes could explain potential, novel, pathological, and molecular mechanisms for childhood asthma and may serve as candidates for therapeutic strategies for this disease.

## Figures and Tables

**Table 1:** Evaluation of each module identified by WGCNA and the module information obtained by hypergeometric algorithm.

ID	Color	Module size	Preservation		Number of DERs	Enrichment	
			*Z*-score	*P* value		Enrichment fold (95% CI)	*P*_hyper_
Module 1	Black	229	6.1362	4.50*E *− 04	14	1.408 [1.146–2.458]	2.51*E *− 02
Module 2	Blue	917	0.8600	1.40*E *− 02	18	0.452 [0.262–0.734]	4.75*E *− 04
Module 3	Brown	482	20.1160	1.80*E *− 30	15	0.716 [0.392–1.218]	2.38*E *− 01
Module 4	Green	307	7.8027	5.00*E *− 03	3	0.225 [0.0458–0.672]	2.50*E *− 03
Module 5	Grey	1274	0.5561	8.10*E *− 12	84	1.518 [1.161–1.969]	1.82*E *− 03
Module 6	Magenta	155	3.4154	6.70*E *− 01	1	0.149 [0.00373–0.848]	2.16*E *− 02
Module 7	Pink	186	23.9128	2.40*E *− 20	9	1.114 [0.496–2.198]	7.15*E *− 01
Module 8	Purple	140	4.8837	6.60*E *− 01	1	0.165 [0.00412–0.940]	3.09*E *− 02
Module 9	Red	302	4.1897	1.50*E *− 01	1	0.0763 [0.00192–0.432]	1.06*E *− 04
Module 10	Turquoise	1270	26.9735	4.90*E *− 125	38	0.689 [0.473–0.979]	3.35*E *− 02
Module 11	Yellow	311	14.4093	4.90*E *− 14	58	4.293 [3.096–5.881]	2.20*E *− 16

**Table 2:** Kyoto Encyclopedia of Genes and Genomes (KEGG) pathways on differentially expressed mRNAs in the coexpressed network.

Term	Count	*P* value	Genes
hsa04512: ECM-receptor interaction	3	9.67*E *− 03	*VWF, LAMB3, LAMA4*
hsa04510: focal adhesion	4	1.00*E *− 02	*VWF, LAMB3, LAMA4, CAV1*
hsa00410: beta-alanine metabolism	2	2.38*E *− 02	*ALDH1A3, SMOX*
hsa04151: PI3K-Akt signaling pathway	4	3.27*E *− 02	*VWF, LAMB3, GNG4, LAMA4*
hsa05200: pathways in cancer	4	4.26*E *− 02	*LAMB3, GNG4, LAMA4, PPARG*

## Data Availability

The datasets used and/or analyzed in this study are available from the corresponding author upon reasonable request.
